# Organs, Cultivars, Soil, and Fruit Properties Affect Structure of Endophytic Mycobiota of Pinggu Peach Trees

**DOI:** 10.3390/microorganisms7090322

**Published:** 2019-09-05

**Authors:** Fei Ren, Wei Dong, Dong-Hui Yan

**Affiliations:** 1 Experimental Center of Forestry in North China, Chinese Academy of Forestry, Beijing 102300, China; 2 China Electric Power Research Institute, Beijing 100192, China; 3 The Key Laboratory of Forest Protection Affiliated to State Forestry Administration of China, Institute of Forest Ecology, Environment and Protection, Chinese Academy of Forestry, Beijing 100091, China

**Keywords:** endophytic fungi, community diversity and structure, Pinggu peach trees

## Abstract

Pinggu peach (*Prunus persica* (L.)) has great economic and ecological value in north China. As a plant, the peach is naturally colonized by a variety of endophytic fungi, which are very important for tree growth and health. However, the mycobiota composition and their affecting factors of the peach trees are still unknown. In our study, the fungal communities in flowers, leaves, stems, and roots of the three cultivars (Dajiubao, Qingfeng, and Jingyan) of Pinggu peach trees and in the rhizosphere soils were investigated by both Illumina Miseq sequencing of ITS rDNA and traditional culturing methods. For organs, except for roots, flowers had the highest fungal richness and diversity, while the leaves had the lowest richness and diversity. Ascomycota and Basidiomycota were the most abundant phyla among samples. The fungal assemblage composition of each organ was distinctive. Fungal communities of the three cultivars also differed from each other. The fungal community structure significantly correlated with soil pH, soil K, fruit soluble solid content, and fruit titratable acidity with the redundancy analysis (RDA). Most isolated fungal strains can be found within high-throughput sequencing identified taxa. This study indicates that plant organs, the cultivars, the soil, and fruit properties may have profound effects on the endophytic fungal community structure associated with Pinggu peach trees. With this study, microbiota-mediated pathogen protection and fruit quality promotion associated with peach trees could be further studied.

## 1. Introduction

Peach naturally hosts a reservoir of endophytic fungi without causing visible disease [1,2]. The endophytic fungi play a vital role in protecting the plant against pathogens, enhancing the natural plant defenses [3,4], and promoting plant growth [5,6,7,8]. Furthermore, they even alter host phenotype and gene expression [9]. For microbial study, culture-independent approaches such as high-throughput sequencing can give huge microbial data and detect unculturable species at a much lower price [4,10,11,12]; however, culture-dependent methods are necessary to get strains for further study and application [4,10,13]; therefore, the two methods should be jointly applied for fungal studies [4,10].

Pinggu peach, produced from Pinggu District of Beijing—China’s peach hometown—is one of the Chinese National Products of its Geographic Identification brand [14]. Pinggu Peach has a serious of excellent cultivars, and is known for its quality and flavor. Peach farming is prevalent in the district and generates more than 1.5 billion RMB for local people and government [14], showing great ecological and economic value. Presently, some peach diseases, i.e., peach-brown rot and peach gummosis disease, seriously threaten the yield of Pinggu peach [15], calling for the development of effective protective measures against a broad range of pathogens and host plants. Studies on endophytes of the peach trees might provide novel pathogen biocontrol and quality-promotion agents. Reports have paid much more attention to fungal endophytes from agricultural crops [16,17], medicinal plants [3,18], and forest trees [19,20]. Relatively, the documentations on the endophytes from fruit trees are still very limited [21,22], including apple and kiwifruit [23,24], wild bananas [25], sour cherry [21], and plums [26]. Few studies have considered peach mycobiota; one study reported that nineteen species were considered to be resident on peach twigs and six were resident on peach flowers in Spain [27]. However, information on endophytic fungi of Pinggu Peach trees is still unavailable.

The peach endophytic community structure could be influenced by both biotic and abiotic factors, such as environmental conditions, the host genes, the interactions among plant microbiota, and geographic differentiation etc. [3,4,28]. The endophytic mycobiota composition of fruit trees can be diverse according to the tree species or even different rootstock/scion combinations [19,29]. While factors affecting endophytic community of Pinggu peach trees is unknown, we speculate that the fungal communities of different organs and cultivars of Pinggu peach trees could be different. Moreover, due to the peach’s special quality and flavor, the community structure may have correlations with the local soil and the fruit’s properties. In this study, the compositional differences of the endophytic mycobiota of different plant organs (flower, leaf, stem, and root) of three cultivars will be investigated. Correlations between the fungal community structure, the cultivars, the soil, and fruit properties will also be researched. With the research results and cultivated fungal strains, further exploitation and utilization for microbiota-related disease resistance, fruit quality, and flavor promotion can be achieved. 

## 2. Materials and Methods

### 2.1. Collection and Processing of Samples

The sampling sites were located in Pinggu District, Beijing (40°00′13”N, 117°00′12”E), where Pinggu Peaches are mainly produced. The three orchards were chosen, each with one cultivar: cultivar1: Dajiubao (T1), Qingfeng (T2), Jingyan (T3). The samples were collected from flowers, leaves, stems, roots, and rhizosphere soils in mid-April 2018. In each orchard, nine peach trees at least 200 m apart were selected randomly. Rhizosphere soil samples were obtained from the soil adhering to tree roots. Organs and soils were evenly mixed, and four biological replicates were chosen for each sample. Samples were collected in sterile plastic bags and processed within 24 h. After washing with tap water, organs were surface-disinfected by ordered washing with 75% ethanol for 1 min, 2% sodium hypochlorite for 3 min, 75% ethanol for 1 min, rinsing in sterile distilled water for 0.5 min, followed by drying [30]. For traditional cultural-dependent isolation, after being surface-disinfected, organs were cut into small pieces (ca. 5 mm × 5 mm) and inoculated in Petri dishes of malt extract agar (MEA) [13]. Petri dishes were sealed, incubated at 25 °C, and examined periodically. A negative control with disinfected plant organs placed on MEA was set up to observe the growth of any externally adhered fungi and to test sterilization efficiency. Soil pH was determined with a glass electrode by stirring the soil suspensions in demineralized water with a ratio of 5 g soil and 25 mL water. Concentrations of soil carbon (C), nitrogen (N), phosphorus (P), potassium (K), and sodium (Na) were tested according to previous methods [31]. The fruit properties of three cultivars (single fruit weight, soluble solids content, titratable acidity) were tested by the standard method [32].

### 2.2. DNA Extraction, Amplification of ITS rDNA Region and Sequencing

Total genomic DNA of the organs as well as isolation strains was extracted with a standard cetyl–trimethyl ammonium bromide (CTAB) method described in Chang et al. [33]. TIANamp Soil DNA Kit (TIANGEN Biotech Co. Ltd., Beijing, China) was used to extract DNA from homogenized soil samples according to the manufacturer’s instructions. The concentrations of the DNA were measured with a NanoDrop ND-2000 spectrophotometer (Thermo Fisher Scientific, Waltham, MA, USA).

Fungal primers ITS1F (CTTGGTCATTTAGAGGAAGTAA) and ITS2R (GCTGCGTTCTTCATC GATGC) were used to amplify the ITS1 region of rDNA [34]. The PCR products were purified and sequenced with an Illumina MiSeq PE300 platform at Shanghai Majorbio Science and Technology Ltd. (with 300 bp length and pair-end reads). The ITS region was amplified using primer pairs ITS1 and ITS4 for cultured strains [35]. High-throughput sequences were deposited at the Sequence Read Archive (SRA) of the National Center for Biotechnology Information (NCBI) under project accession number PRJNA551780. Sequences of isolated stains were under NCBI no. MN339608-MN339658 and MN340269-MN340273.

### 2.3. Data Processing and Analysis

Sequence quality filtering on the raw reads was performed using FLASH [36] and Trimmomatic [37]. Mothur standard operation pipeline (SOP, v.1.37.6) [38] was used to analyze the data and classify sequences into operational taxonomic units (OTUs) at 97% similarity against UNITE Database v. 7.2 [39]. Sequence reads were subsampled for each sample with the minimum number of reads among all samples before comparative analysis. The species richness (Sobs), diversity (Invsimpson), and evenness (Simpsoneven) [40] were calculated in Mothur. Data for rarefaction curves were also generated in Mothur. One-way ANOVA (analysis of variance) was used to identify differences in community richness, diversity, and evenness. The R language platform [41] was used for analysis and visualization of the data sets of the microbial diversity and abundances in different samples (rarefaction curves, Venn, bar chart, PCoA, RDA, PERMANOVA). LEfSe analysis [42] was used to find taxa significantly affecting sample structure at different levels with an LDA threshold of two and an all-against-all strategy. The sequences of cultured strains were analyzed and identified by NCBI blast [43].

## 3. Results

### 3.1. Overview of the Sequencing Data and Alpha Diversit of Fungal Communities

After quality control, 3,866,840 high quality sequences were obtained and classified into 2158 OTUs (excluding singletons). The number of sequences in each sample ranged from 45,672 to 73,649 with an average of 64,447 ± 8918 (mean ± SD) sequences. The average sequence length was 234 bp. The Alpha-diversity indices are shown in Table 1. For organs, the roots had the highest fungal richness (310.67) and diversity (10.30), followed by flowers, and the lowest richness and diversity was found in leaves (108.25 and 4.08). The flowers had the highest evenness (0.082), while the lowest evenness was observed in roots (0.034). Fungal Alpha-diversity indexes of tissues and soils showing statistically significant differences are shown in Figure S1 labeled with asterisk (*). The rarefaction curve indicated that the number of OTUs was sufficient and saturated in each sample (Figure S2).

### 3.2. Fungal Composition and Relative Abundance among Different Organs and Soils

**Figure 1:**
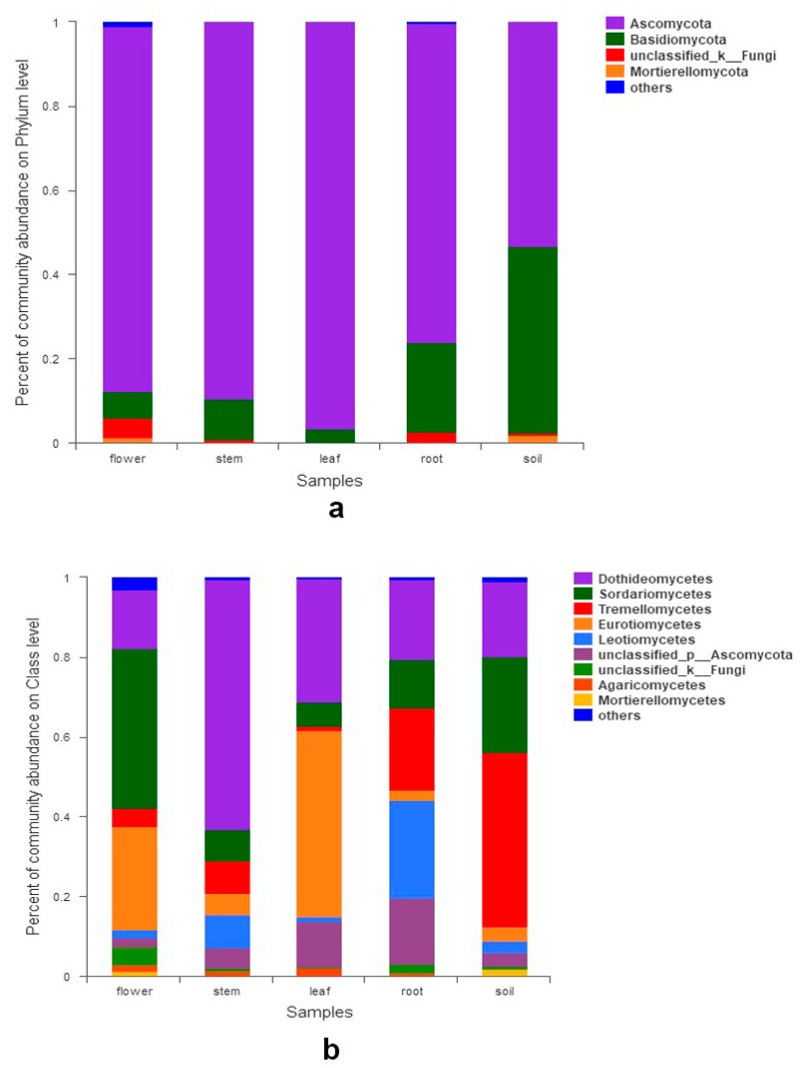
Fungal abundance in different organs and soil: (**a**) at phylum level; (**b**) at class level.

**Figure 2:**
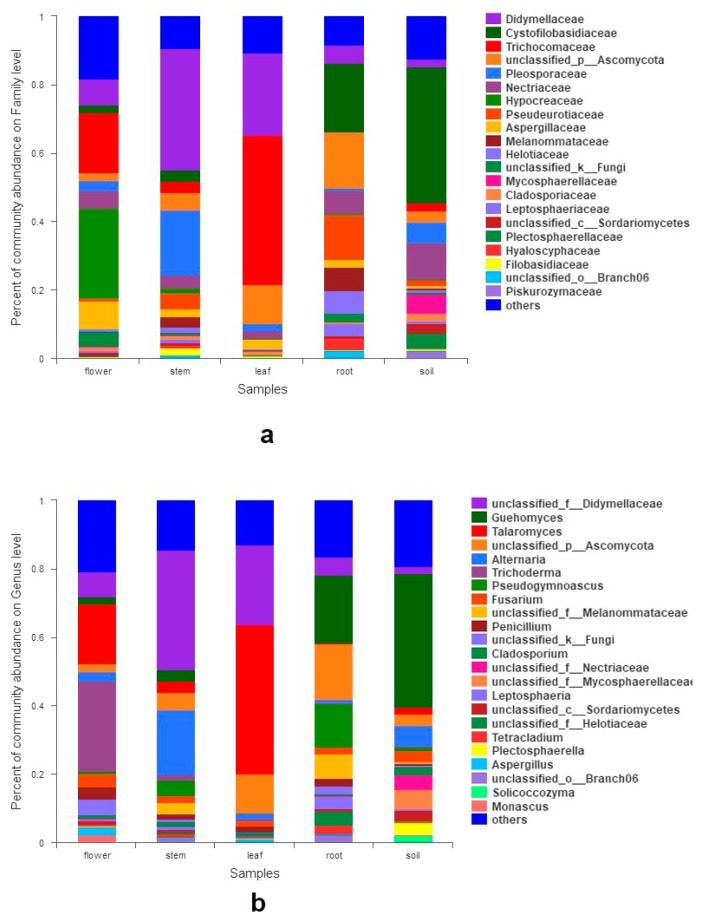
Fungal abundance in different organs and soil: (**a**) at family level; (**b**) at genus level.

**Figure 3:**
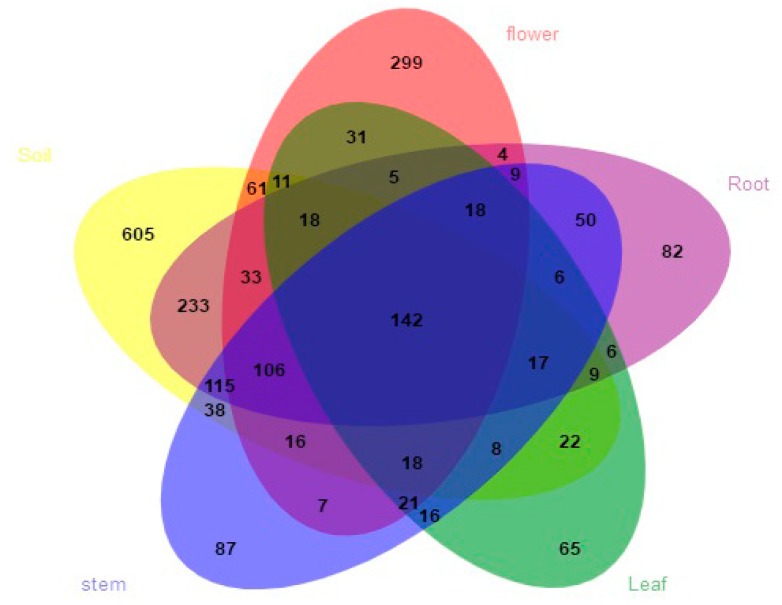
Venn diagram showing shared and unique fungal operational taxonomic units (OTUs) in each organ (flower, leaf, stem, root) and soil.

We totally detected seven phyla, 42 classes, 105 orders, 249 families, and 497 genera. At the phylum level, Ascomycota was the most abundant group, followed by Basidiomycota. Mortierellomycota, Chytridiomycota, and Kickxellomycota were much less frequent (< 0.1%). The relative abundances of phyla exceeding 1% in each organ are shown in Figure 1a. Seven classes had a relative abundance of more than 1% (Figure 1b), which include Dothideomycetes, Sordariomycetes, Tremellomycetes, Eurotiomycetes, Leotiomycetes, Agaricomycetes, and Mortierellomycetes. Dothideomycetes predominated in stems and their sequences were the most abundant of all. At the family level, the relative abundance of 17 families exceeded 2% (Figure 2a). Abundance varied among samples, i.e., Hypocreaceae (Sordariomycetes) were most abundant in flowers, while Trichocomaceae (Eurotiomycetes) were richest in leaves. Fourteen fungal genera had a relative abundance of more than 2% (Figure 2b), such as *Guehomyces*, *Talaromyces*, *Trichoderma*, *Alternaria*, *Fusarium*, *Penicillium*, *Aspergillus*, and *Cladosporium*. Only a few species were identified, such as *Guehomyces pullulans* (Lindner) Fell & Scorzetti, *Aspergillus subversicolor* Jurjević, S.W. Peterson & B.W. Horn, *Penicillium raperi* G. Sm., and *Talaromyces pinophilus* (Hedgc.) Samson, N. Yilmaz, Frisvad & Seifert. Most sequences could only be classified at the genus or higher level. The sampled organs and soils shared 142 (6.6%) of the total 2158 OTUs. The proportion of OTUs unique to a certain organ ranged from 3.0% (65 OTUs; leaves) to 13.8% (299 OTUs; flowers, belonging to genera *Corniculariella*, *Glaciozyma*, *Xylaria*, *Pluteus*, *Macrophoma*, *Mycena* etc.) (Figure 3). LEfSe analysis demonstrated the relative abundance of fungi at varied taxonomic levels. Significantly different taxa from phylum level to genus level are presented in Figure S3A,B. For example, in flowers, the abundance of Sordariomycetes, Hypocreales, Aspergillaceae, and *Aspergillus* affected the structure differences the most, while Ascomycota, Eurotiomycetes, Eurotiales, Trichocomaceae, and *Talaromyces* contributed the most to leaf microbial structure differences (Figure S3B).

### 3.3. Fungal Community among Three Peach Cultivars

**Figure 4:**
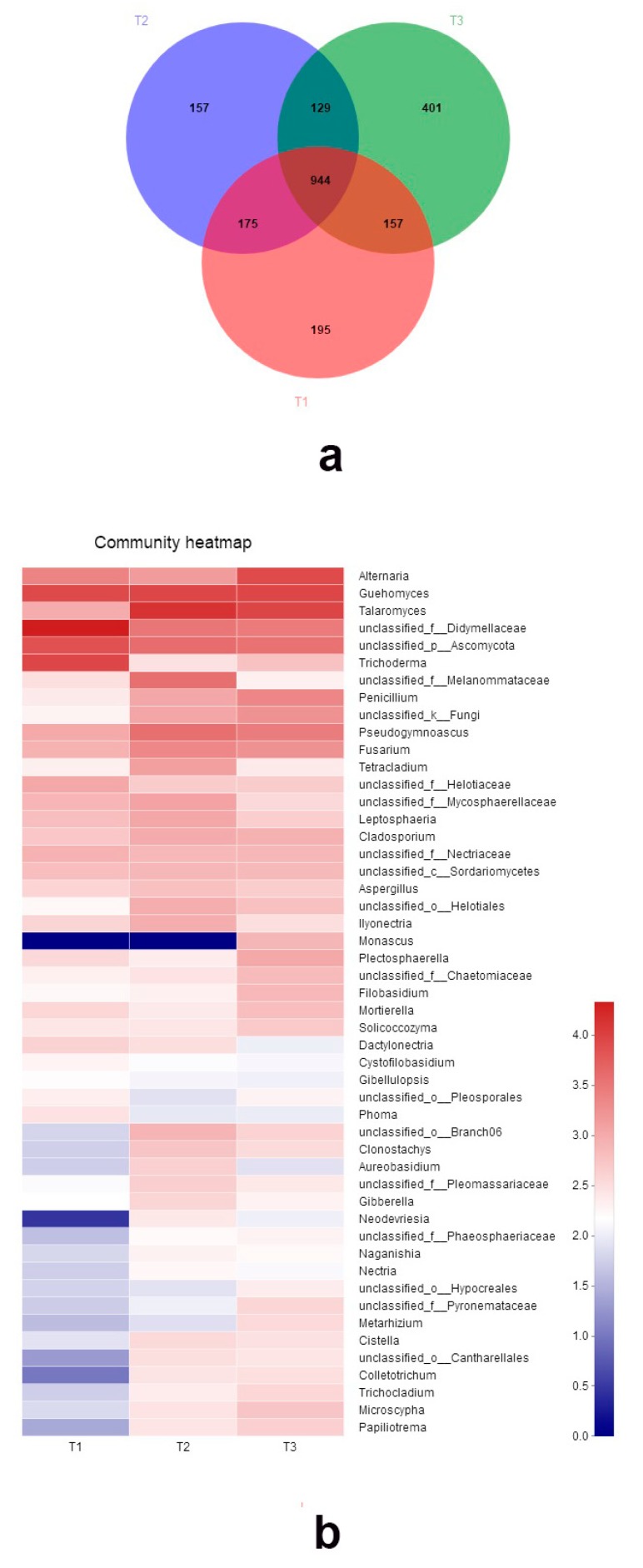
Fungal community among three peach cultivars: (**a**) Heatmap of the top 50 genera of three cultivars; (**b**) Venn diagram showing shared and unique fungal OTUs of three Pinggu peach cultivars.

The three cultivars shared 944 (43.7% of the total) OTUs. The OTUs unique to the cultivars comprised 195, 157, and 401 OTUs for T1, T2, and T3, respectively (Figure 4a). The fungal composition of three cultivars varied. Ascomycota was also the most abundant phyla. Since many sequences cannot be identified to species, here, the relative abundance of the top 50 genera is shown in the heatmap of Figure 4b. Taxa abundance significantly differed among three cultivars, as can be found in Figure S4A–D. The abundance of five genera—*Alternaria*, *Talaromyces*, *Trichoderma*, *Fusarium*, and *Penicillium*—all showed significant difference among three cultivars.

### 3.4. Impacts of Organs, Peach Cultivars, Soil Properties, and Peach Fruit Properties on Fungal Community Structure 

**Figure 5:**
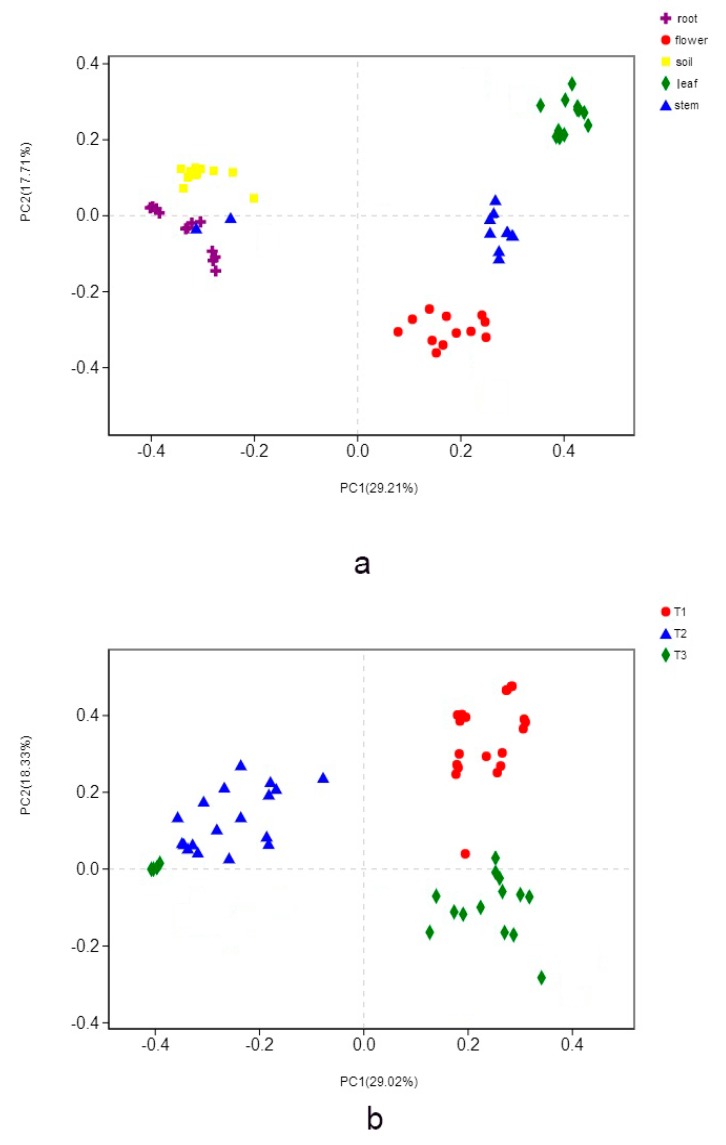
Principal coordinates analysis (PCoA) based on the relative abundance of fungal OTUs showing the fungal community structure: (**a**) in different organs and soil of the Pinggu peach trees; (**b**) in three different cultivars.

The organs and cultivars affected the fungal population and the comparison using principal coordinate analysis (PCoA) revealed differences between communities: each organ and soil formed individual cluster (Figure 5a); three cultivars also formed different fungal clusters (Figure 5b). PERMANOVA test confirmed the significant differences in community structures among the organs and the soils (*p* <0.05 in all possible pairs) as well as the three cultivars (*p* < 0.05).

**Figure 6:**
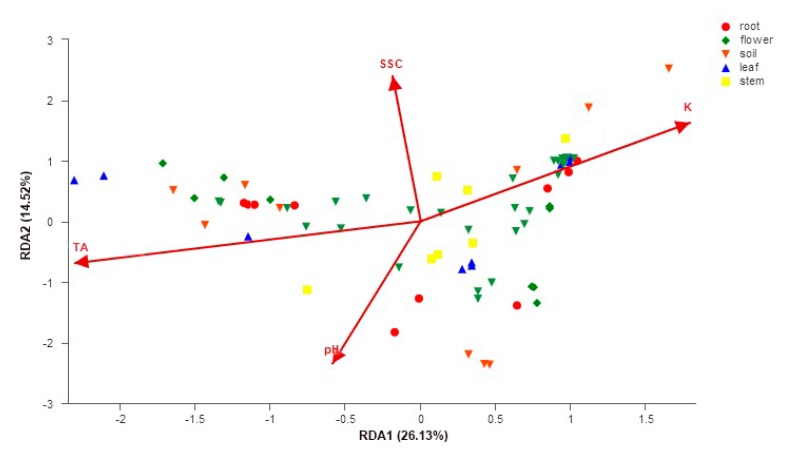
Redundancy analysis (RDA) plot showing the correlation between fungal community structure and soil properties and peach fruit properties. SSC—soluble solid content, TA—titratable acidity.

The RDA tests showed that the fungal community structure was significantly correlated with soil pH (*R^2^* = 0.78, *p* = 0.001), soil K (*R^2^* = 0.83, *p* = 0.001), fruit soluble solid content (SSC) (*R^2^* = 0.77, *p* = 0.001), and fruit titratable acidity (TA) (*R^2^* = 0.79, *p* = 0.001) (Figure 6). The soil properties of three orchards and peach fruit properties are listed in Supplementary Tables S1 and S2.

### 3.5. Culture-Dependent Isolation of Endophytic Fungi of the Pinggu Peach Trees

Fifty-six strains were isolated using traditional disk methodology (Table 2). Genera generally belong to *Penicillium*, *Fusarium*, *Alternaria*, *Talaromyces*, *Trichoderma*, and *Cladosporium*. Strains belonging to *Meyerozyma*, *Arthrinium*, *Chaetomium* were also cultured. Numbers in parentheses indicate duplicated strains isolated.

## 4. Discussion

It has been reported that Ascomycota fungi have higher species diversity due to their faster evolutionary rate and adaptability [44]. This may partially explain our result that the fungal communities were predominated by Ascomycota in all the samples and previous studies that plant mycobiota was mainly consist of Ascomycota, and then Basidiomycota [4,10,21]. The Class Dothideomycetes was the most abundant class in stems. It was also the largest group in *Pinus halepensis* Mill. [45], *Lycopodium annotinum* L., and *Lycopodium clavatum* L. [46], blackcurrant berries of Lithuania [28], and Jingbai pear of China [30]. The majority of the class were found to be endophytes, saprobes growing on woody debris, decaying leaves or dung, as well as several plant pathogens [20,46,47]. Sordariomycetes were most abundant in flowers, and have the greatest impact on the mycobiota structure of flowers. Their members include endophytes, saprobes, and coprophilous, fungicolous, and lichenicolous taxa [48,49]. The same condition is found of Eurotiomycetes in leaves. Eurotiomycetes members can be animal and plant pathogens, endophytes, mutualistic forming ectomycorrizae, and lichens, and are used in food production [50]. Given the high abundance and significance, the two classes can be considered as the key classes (Sordariomycetes for flowers, Eurotiomycetes for leaves). The high abundance of these two classes in Pinggu tree flowers and leaves could also indicate their active role in the two organs, respectively.

The Aspergillaceae family affected the fungal structure differences the most in flowers, while Trichocomaceae had this affect in the same conditions in leaves. Aspergillaceae, including members of *Aspergillus* and *Penicillium*, two genera with a close relationship and wide-spread in soils and plants, can often be found on oranges and other fruit, and are used in vine and vinegar making [51]. Fumimycin produced by *Aspergillus* is a target in antibacterial, antimalarial, and anticancer drug discovery [52]. Members of Trichocomaceae occur commonly and are important to both industry and medicine, associated with food spoilage and mycotoxin production. Some species are opportunistic pathogens, while others are exploited in biotechnology for the production of enzymes, antibiotics, and other products [53]. Apart from *Penicillium* and *Aspergillus*, *Talaromyces*, *Trichoderma*, *Alternaria*, *Fusarium*, and *Cladosporium* are also abundant. *Talaromyces* spp. are widely distributed and can participate in leaf litter decomposition [54]. Strains from mangrove forests had bioactive effects of secondary metabolites, especially cytotoxic/antiproliferative activity against tumor cell lines, antimicrobial effects, and immunosuppressive and enzyme inhibitory aptitudes [55]. *Talaromyces* spp. were also reported to be very abundant from fruit cherry [21], apple trees [22], and Jinbai pear [30]. *Trichoderma* spp. are free-living fungi which are common in soil and root ecosystems. They are opportunistic, avirulent plant symbionts, as well as being parasites of other fungi [56]. Root colonization by *Trichoderma* spp. enhances root growth and development, crop productivity, resistance to abiotic stresses, and the uptake and use of nutrients [55]. *Alternaria* species are ubiquitous in the environment and are involved in saprobic, endophytic, and pathogenic species with a large variety of substrates [57]. They can produce highly bioactive metabolites, such as isobenzofuranone A and indandione B, showing significant inhibitory activities against tumor cell lines [58]. Some members show antimicrobial activities and have great potential for biological control of plant diseases [59]. *Fusarium* has a wide distribution in soil and plants, is one of the most important groups of plant pathogenic fungi, and affects a huge diversity of crops across the globe [60]. *Cladosporium* are cosmopolitan in their distribution and are commonly encountered on all kinds of plant, fungal, and other debris [61]. They are frequently isolated from soil, food, paint, textiles, and other organic matters or colonize leaf lesions caused by plant pathogenic fungi as secondary invaders [61].

### 4.1. Fungal Communities among Organs

Previous reports have shown that different plant organs host different fungal communities [11,20,28]. The findings are consistent with the results in this study, since each organ forms a distinctive microenvironment and is spatially distant [4,20,32]. As we know, for most plants, flowers are the basis of subsequent fruits. It is worth noting that flowers had the highest fungal richness and diversity except for roots. Flowers had the most unique OTUs in our study. Sordariomycetes, Hypocreales, Aspergillaceae, and *Aspergillus* mostly determined the mycobiota structure differences of the flower tissues. Due to their ephemerality and exquisite anatomy, flowers provide unique habitats to microorganisms, including a range of distinct microscale niches [62]. Shade et al. [63] suggested that changes in apple flower microbial community structure are predictable over the life of the flower, providing a basis for ecological understanding and disease management. However, more studies have paid attention to below-ground parts (roots and soils) of plants [4]; therefore, the flower microbiome is more poorly understood. Studies concerning community composition and diversity of the flower microbiota, spatial and temporal community dynamics, and the interactions between flower microbes, plant hosts, and pollinators should be conducted in the future [62]. 

### 4.2. Fungal Communities of Cultivars

The three different cultivars harbored unique fungal communities. Several studies have reported similar results [3,63]. Fungal communities of olive cultivars showed significant varied fungal richness and composition associated with the presence of olive pathogens causing leaf spots [64]. Two peach cultivars have different capacities for disease resistance, and with different bacterial communities, increased proportions of antagonistic bacteria might contribute to the natural defense of the resistant cultivar [65]. Regarding bahiagrass in the USA, the influence of cultivar selection on soil fungal and bacterial communities is low, although specific taxa appeared to be cultivar-dependent, which may has implications for the control of plant pathogens [66]. Microbial differences of cultivars could be determined by host genetic background differences.

### 4.3. Correlation between Fungal Communities and Soil and Fruit Properties 

Fungal community structure was significantly correlated with soil (soil pH and K content) and fruit properties (fruit soluble solid content and titratable acidity). Plants and soils, especially rhizosphere soil, have a very tight mutual relationship. Plant roots absorb water and nutrients from soil and secrete organic exudates; rhizosphere soil provides water and nutrients for the plant and regulates plant properties [4,67]. Rhizosphere surrounding plant roots is estimated to contain millions of microorganisms, and scientists consider it to be a highly complex and dynamic ecosystem; the plant gut microbiome [67]. Soil type and properties may even alter root development and root exudation [68]. In our recent study, the endophytic fungal communities associated with Jingbai pear trees were also significantly related to soil properties [30]. It is not difficult to understand that soil properties may have an influence on the fungal community structure associated with the peach trees. The producing area of Pinggu peaches is unique. The geographic location (large number of potassium-rich volcanic rocks in surrounding mountains) results in distinctive soil properties, and the soils, water, and sunshine in the area results in the distinctive quality and flavor of the fruit (big fruit, sweet, and less sour) [14]. Studies have shown the important role of microbes in fermentation and flavor of fruit [69] and freshly prepared juices [70]; *Alternaria*, *Aspergillus*, *Cladosporium*, and *Fusarium* were observed in tested juice samples [70]. These genera numbers were also found in our study. Their functional role with peach fruit and relations between fruit microbiota with fruit properties merits study in the future. 

### 4.4. Isolated Endophytic Strains and Future Studies

Combined use of culture-dependent and culture-independent approaches is a useful method for microbial studies [4,10,13]. To the best of our knowledge, this is the first investigation of the endophytic mycobiota in Pinggu peach trees with implementation of both PCR-based Illumina next-generation sequencing technology and traditional culture methodology. Many studies of peach-related microbes have focused on pathogens [71], arbuscular mycorrhizal fungi [72], and some bacteria [65,73]. Few have dealt with peach mycobiota; one concerned mycoflora of peach twigs (19 species) and flowers (six species) in Spain [27]. Our study gives a comprehensive picture for the unexplored fungal diversity of Pinggu peach trees. These results also provide valuable reference for studies of fungal communities of other peach trees.

Following isolation, genera were generally *Penicillium*, *Fusarium*, *Alternaria*, *Talaromyces*, *Trichoderma*, *Cladosporium*. Their existence was verified by Illumina sequencing. Nowadays, high-throughput sequencing has the great advantage of generating huge species data at an affordable price [4,10,11,12]. Isolated stains are limited by traditional methods in our study. High-throughput culturing of fungi from plants by the dilution-to-extinction technique to generate large numbers of fungal extinction cultures, and coculture with pathogens to study the strains’ inhibitory abilities could be done as a next step [74]. The plant microbiome highlights the importance of the endospheric microbiome for growth and health of host plants. Microbial community analysis represents an elegant way to identify keystone microbial species holding central positions in the community [75]. A recent study compared the endophytic bacterial and fungal community in banana roots and shoot tips during growth and wilting processes, and accessed the interactions between the keystone species and plants during the *Fusarium* wilt process [75]. The keystone species were isolated and further engineered to improve banana wilt resistance [75]. The most abundant plant-associated microbes can be isolated and maintained axenically [75,76], which opens new avenues to systematically screen for desirable traits using microfluidic systems, high-throughput screens, or microbiota reconstitution experiments with SynComs (synthetic microbial communities) and germ-free plants [77]. The design of SynComs based on traits involved in microbiota-modulated immunity (MMI) and/or direct microbial competition (DMC) represents a promising direction to achieve robust plant protective activities against pathogens [78]. 

## 5. Conclusions

In conclusion, each organ (flower, leaf, stem, and root) hosted a different fungal assemblage. Ascomycota and Basidiomycota were the most abundant phyla. Fungal communities of the three cultivars also varied. The structure of the fungal community remarkably correlated with soil pH, soil K, fruit soluble solid content, and fruit titratable acidity. Most isolated cultures were included in the taxa identified by high-throughput sequencing. Plant organs, the cultivars, the soil, and fruit properties might affect the endophytic microbial community structure associated with Pinggu peach trees. Combining the next-generation sequencing results and cultivated endophytic fungal strains to systematically screen and the design of SynComs for microbiota-mediated pathogen protection and fruit quality promotion is worthy of further study.

## Figures and Tables

**Table 1:** Richness, diversity, and evenness indexes of fungal communities (mean ± SD).

Samples	Sobs (Richness)	Lnvsimpson (Diversity)	Simpsoneven (Evenness)
leaf	108.25 ± 1.84	4.08 ± 0.11	0.041 ± 0.001
flower	207.83 ± 16.16	7.86 ± 1.27	0.082 ± 0.006
stem	176.92 ± 4.81	7.81 ± 0.59	0.041 ± 0.002
root	310.67 ± 2.84	10.30 ± 0.15	0.034 ± 0.001
soil	627.41 ± 13.94	8.46 ± 0.31	0.014 ± 0.002

**Table 2:** Isolation of endophytic fungi of the Pinggu peach trees.

Sample Number	Identification	NCBI Number	Percent of Identity
DH2 (5)	*Penicillium funiculosum* isolate B12	JN676119	99%
DGE1 (4)	*Trichoderma hamatum* strain 347	KX357867	99%
DGE3 (6)	*Fusarium solani* isolate C10-4	KT876641	99%
HY1(2)	*Meyerozyma guilliermondi*	KP764945	100%
DY4 (4)	*Talaromyces stollii*	JX965246	100%
HY4 (4)	*Penicillium oxalicum strain* QHBC11	KC880081	100%
DY2 (3)	*Fusarium fujikuroi*	NR_111889	100%
HZ6 (3)	*Alternaria alternata*	NR_131263	99%
DY6 (3)	*Talaromyces stollii*	NR_111781	98%
DH5 (3)	*Penicillium caperatum* CBS 443.7	NR_138333	99%
HY3 (2)	*Chaetomium globosum*	NR_144833	99%
HZ1(2)	*Trichoderma sp.* isolate yi0319	MK326900	97%
HZ4 (3)	*Alternaria solani* isolate OTA52	JF491196	97%
HZ5 (4)	*Aspergillus niger*	NR_111348	99%
DY5 (2)	*Cladosporium cucumerinum*	NR_119841	99%
HY2 (3)	*Alternaria sp.* isolate JS8-5	MF033857	99%
TY6 (3)	*Arthrinium* sp. GU071007	AB471012	99%

## Supplementary Materials

The following are available online at https://www.mdpi.com/2076-2607/7/9/322/s1, Figure S1: Statistically significant differences in the fungal species richness (A), diversity (B), and evenness (C) of the samples, * 0.01 < *p* ≤ 0.05, ** 0.001 < *p* ≤ 0.01, *** *p* ≤ 0.001. Figure S2: Rarefaction curves of all the samples. All the samples are saturated for further study. Figure S3: LEfSe analysis showing the relative abundance of fungi at varied taxonomic levels. (A) Cladogram of different taxa from level phylum to genus, different color nodes represent significantly enriched and important taxa among samples, (B) LDA graph, the higher LDA scores the taxa are, the more effect the taxa have. Figure S4. Significantly different taxa among three cultivars. (A) At phylum level, (B) at class level, (C) at family level, (D) at genus level.
