# Supplementary material for: Organs, Cultivars, Soil, and Fruit Properties Affect Structure of Endophytic Mycobiota of Pinggu Peach Trees
Source: Microorganisms. 2019 Sep 5;7(9):322. doi: 10.3390/microorganisms7090322 (PMC6780621; doi:10.3390/microorganisms7090322)
Supplement: Supplementary file 1 [file microorganisms-07-00322-s001.zip › Sup.Figs/Supplementary Tables.pdf]

Supplementary Table 1 The soil properties of three orchards

Soils properties	Orchard1 (T1)	Orchard 2 (T2)	Orchard 3 (T3)
pH	6.5(±0.1）	6.2(±0.1）	6.8(±0.2）
Soil C(g/kg)	41.4(±0.3）	48.5(±0.2）	49.3(±0.4）
Soil N(g/kg)	3.45(±0.05）	4.39(±0.02）	4.25(±0.02）
P (g/kg)	0.526(±0.004)	0.484(±0.002)	0.583(±0.002)
K (g/kg)	25.4(±0.2）	22.7(±0.20）	26.4(±0.2）
Na (g/kg)	14.0(±0.1）	14.5(±0.2）	13.8(±0.2）

Supplementary Table 2 The fruit properties of three cultivars

Fruit properties	T1	T2	T3
Single fruit Weight (g)	255(±2.5）	190(±2.2）	285(±3.3）
Soluble solid Content (%)	15.5(±0.2）	13.5(±0.2）	14.9(±0.3）
Titratable acidity (%)	0.19(±0.03）	0.29(±0.05）	0.16(±0.02）
