# Supplementary figures and images for: Organs, Cultivars, Soil, and Fruit Properties Affect Structure of Endophytic Mycobiota of Pinggu Peach Trees
Source: Microorganisms. 2019 Sep 5;7(9):322. doi: 10.3390/microorganisms7090322 (PMC6780621; doi:10.3390/microorganisms7090322)

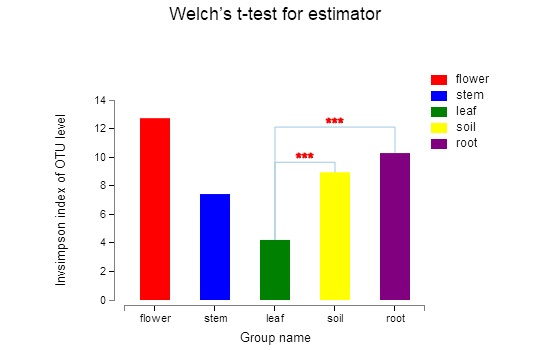

Supplement: Supplementary file 1 [file microorganisms-07-00322-s001.zip › Sup.Figs/Insim.jpg]

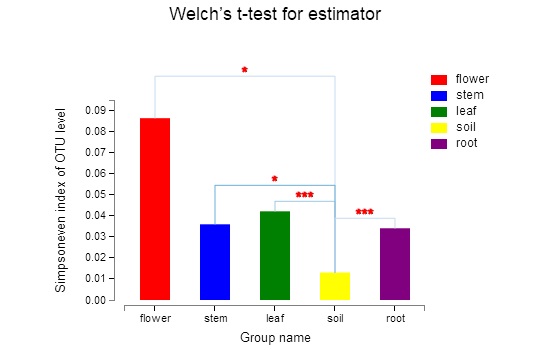

Supplement: Supplementary file 1 [file microorganisms-07-00322-s001.zip › Sup.Figs/Simpeven.jpg]

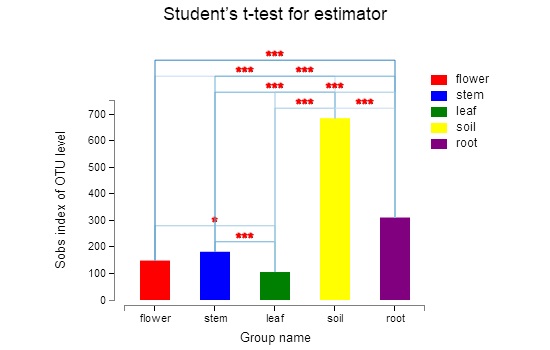

Supplement: Supplementary file 1 [file microorganisms-07-00322-s001.zip › Sup.Figs/sobs differ.jpg]

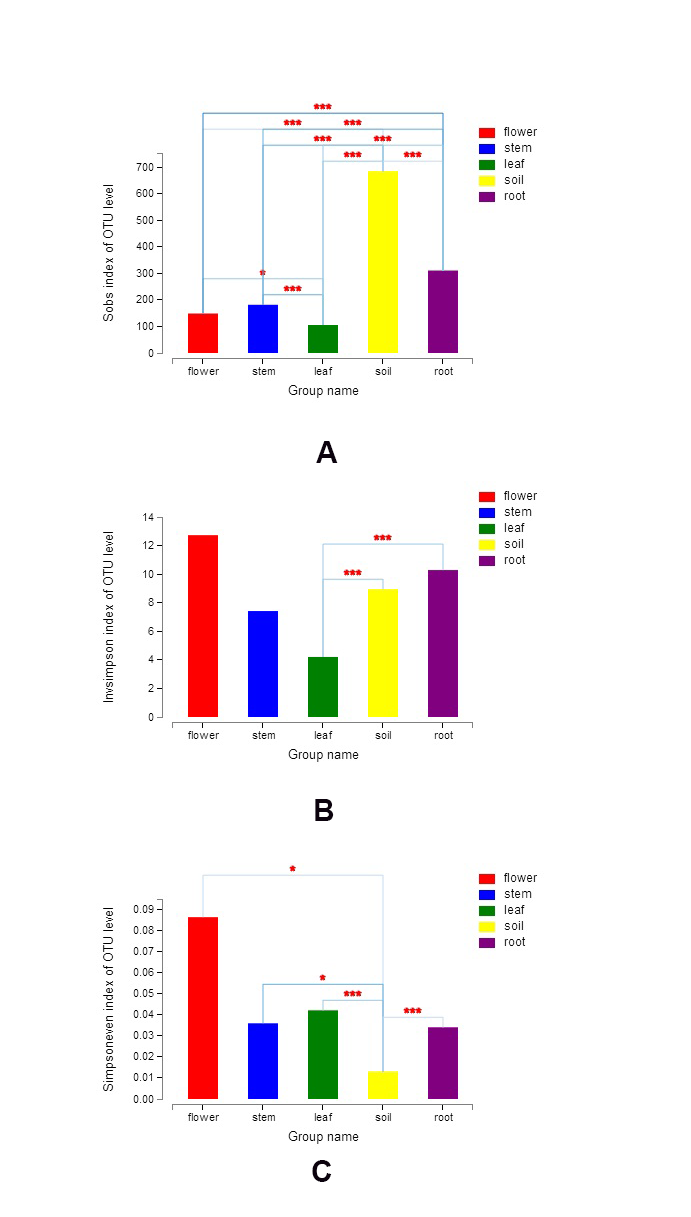

Supplement: Supplementary file 1 [file microorganisms-07-00322-s001.zip › Sup.Figs/Supplementary Fig.S1.tif]

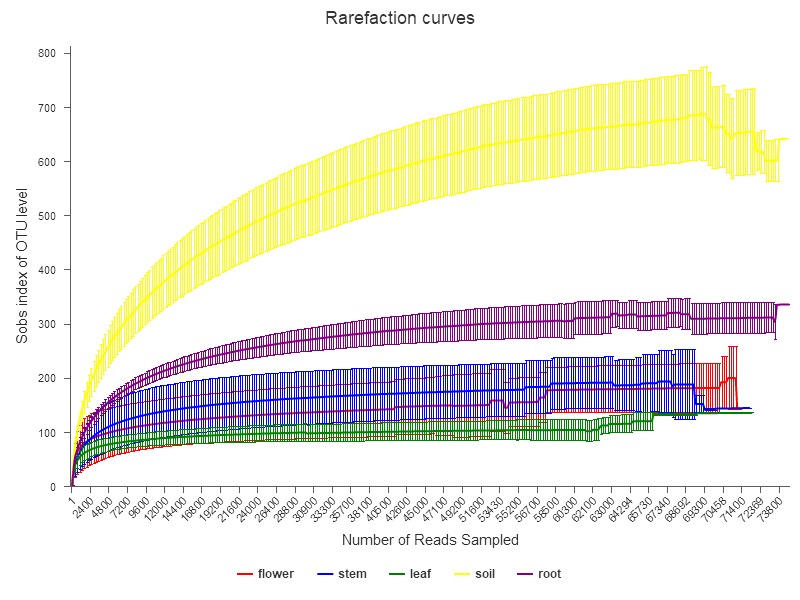

Supplement: Supplementary file 1 [file microorganisms-07-00322-s001.zip › Sup.Figs/Supplementary Fig.S2.jpeg]

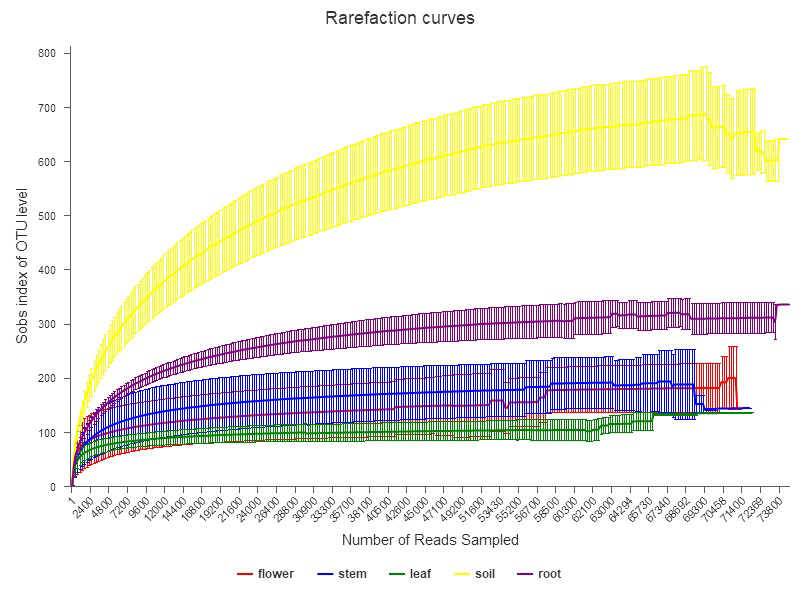

Supplement: Supplementary file 1 [file microorganisms-07-00322-s001.zip › Sup.Figs/Supplementary Fig.S2.tif]

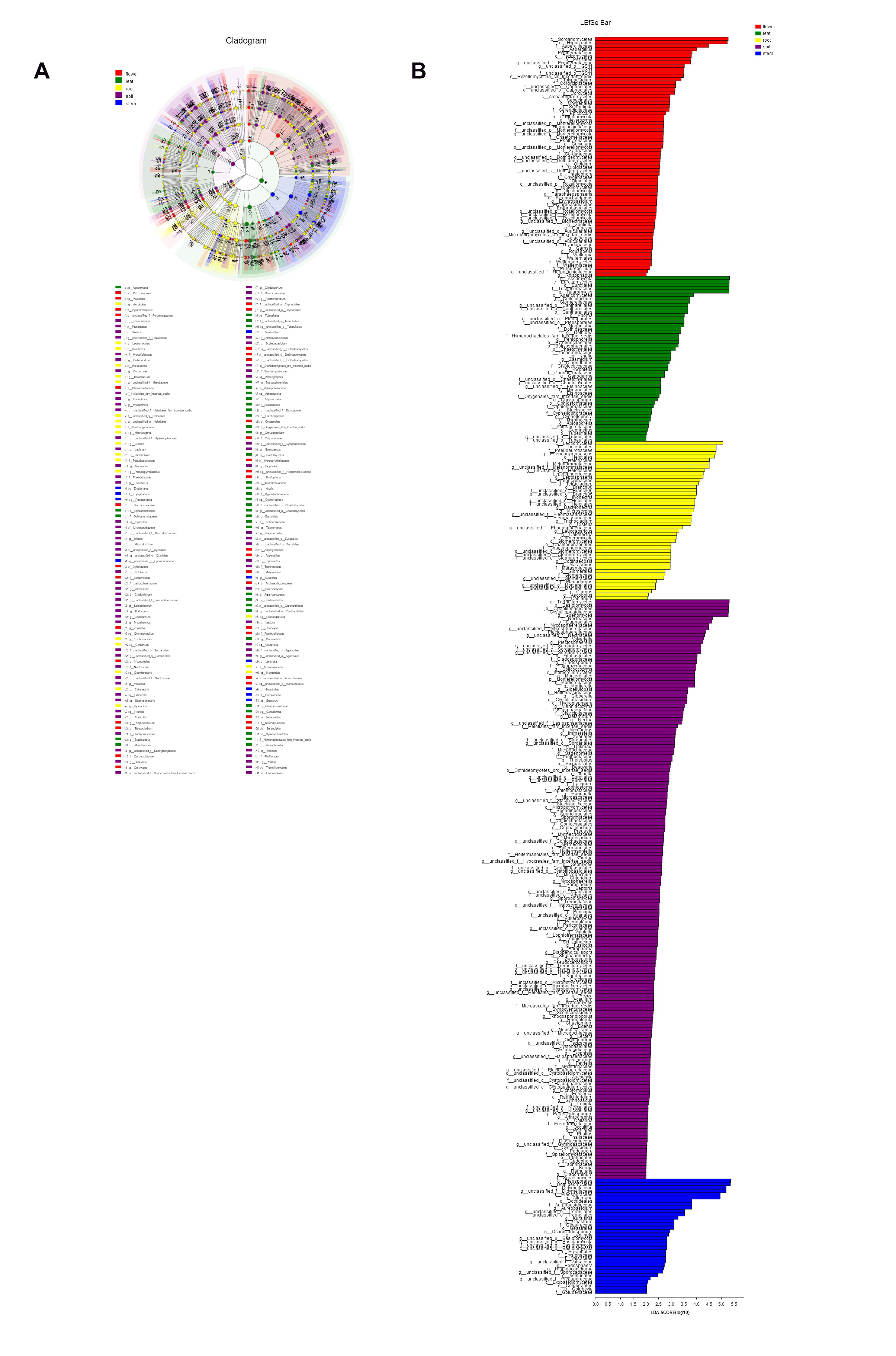

Supplement: Supplementary file 1 [file microorganisms-07-00322-s001.zip › Sup.Figs/Supplementary Fig.S3.tif]

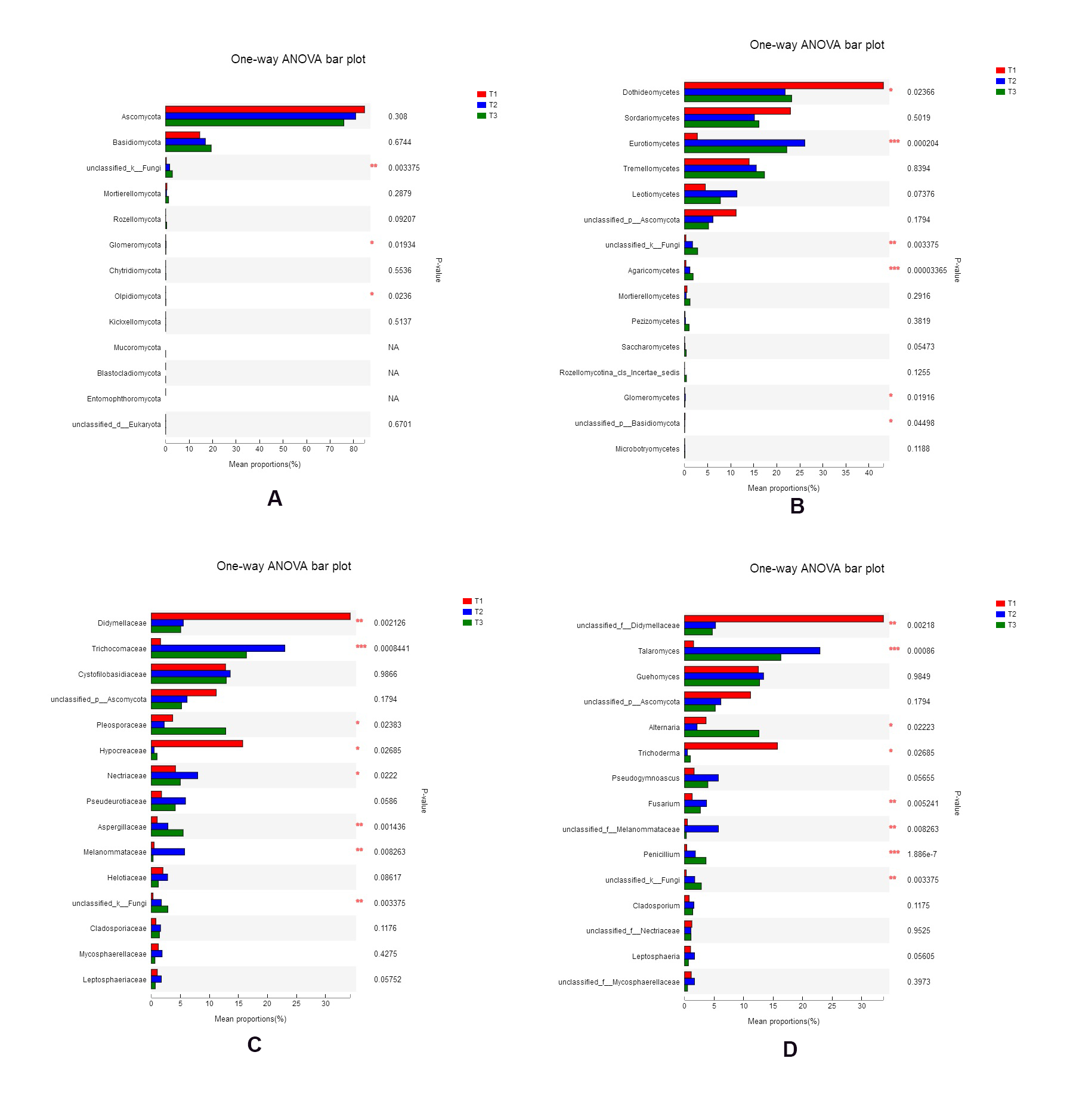

Supplement: Supplementary file 1 [file microorganisms-07-00322-s001.zip › Sup.Figs/Supplementary Fig.S4.tif]
